# A novel reversed phase high performance liquid chromatography approach for stability-indicating simultaneous analysis of brimonidine tartrate and timolol maleate

**DOI:** 10.3389/fchem.2025.1678541

**Published:** 2025-12-01

**Authors:** Aktham Mestareehi

**Affiliations:** 1 Department of Applied Pharmaceutical Sciences and Clinical Pharmacy, Faculty of Pharmacy, Isra University, Amman, Jordan; 2 Department of Pharmaceutical Sciences, Eugene Applebaum College of Pharmacy and Health Sciences, Wayne State University, Detroit, MI, United States

**Keywords:** brimonidine tartrate, timolol maleate, high-performance liquid chromatography (HPLC), specificity, linearity, accuracy, precision, limit of detection (LOD)

## Abstract

**Introduction:**

The simultaneous quantification of Brimonidine Tartrate and Timolol Maleate in ophthalmic formulations is essential for ensuring product quality, efficacy, and stability. However, few methods provide adequate selectivity, sensitivity, and environmental sustainability. This study aimed to develop and validate a robust, rapid, and reproducible reversed‐phase high‐performance liquid chromatography (RP‐HPLC) method with stability-indicating and green chemistry attributes.

**Methods:**

Chromatographic separation was performed on a Supelco Discovery C18 column (25 cm × 4.6 mm, 5 μm) using isocratic elution with phase A (30 mM triethylamine buffer, pH 7.0) and phase B (acetonitrile) in a ratio of 80:20. The flow rate was 1.0 mL/min, and analytes were detected at 245 nm and 295 nm using a diode array detector. Method validation followed ICH Q2 (R1), USP, and FDA guidelines, evaluating linearity, accuracy, precision, specificity, robustness, and sensitivity. Forced degradation under acid, base, oxidative, thermal, and photolytic conditions was conducted to assess stability-indicating capability. Green analytical chemistry (GAC) metrics were calculated using Eco-Scale, GAPI, and AGREE tools.

**Results:**

The method exhibited excellent linearity over 100–500 ppm for Brimonidine Tartrate and 250–1,250 ppm for Timolol Maleate. Accuracy ranged from 99.42% to 99.82% for Brimonidine Tartrate and 98.71% to 101.10% for Timolol Maleate. Precision, specificity, and robustness results showed relative standard deviations below 2%. The LODs were 0.08 ppm for Brimonidine Tartrate and 0.20 ppm for Timolol Maleate, while LOQs were 0.24 ppm and 0.60 ppm, respectively. Forced degradation confirmed the method’s ability to separate both drugs from their degradation products. Brimonidine Tartrate remained stable under all stress conditions, whereas Timolol Maleate was susceptible to hydrolytic and oxidative degradation. The method demonstrated moderate greenness with an Eco‐Scale score of _~_75, a GAPI pictogram with mixed green/yellow zones, and an AGREE score of 0.57.

**Discussion:**

The validated RP-HPLC method proved accurate, precise, sensitive, and stability‐indicating for the simultaneous determination of Brimonidine Tartrate and Timolol Maleate. Its moderate GAC performance supports a balance between analytical rigor and sustainability. These findings establish the method as suitable for routine quality control and stability testing of ophthalmic formulations containing both drugs.

## Introduction

Glaucoma ranks among the leading causes of chronic vision impairment and is recognized as the third most common cause of blindness worldwide (Resnikoff et al., 2004). Elevated intraocular pressure (IOP) remains the primary modifiable risk factor linked to vision loss in glaucoma patients (Bengtsson et al., 2007). Evidence from extensive clinical trials underscores the critical role of promptly lowering IOP to protect against optic nerve damage and preserve visual function (Kass et al., 2002). Importantly, results from the Early Manifest Glaucoma Trial demonstrated that lowering intraocular pressure (IOP) by as little as 1 mmHg is associated with an approximate 10% reduction in the risk of glaucoma progression (Leske et al., 2004; Moisseiev et al., 2013). Initial treatment for glaucoma generally begins with monotherapy using a single medication. If this approach fails to sufficiently lower intraocular pressure (IOP), clinicians may either switch to a different single agent or introduce an additional medication (European Glaucoma Society, 2021). For patients who require multiple drugs to achieve target IOP, fixed-combination therapies are often favored, as they tend to improve adherence to treatment (Gugleta et al., 2003). Interestingly, despite guidelines recommending monotherapy as the starting point, real-world practice shows that many patients with primary open-angle glaucoma or ocular hypertension are started directly on combination therapy (Crichton et al., 2010; Mestareehi, 2024).

Combigan® (Allergan Inc, Irvine, CA, United States) (Combigan®, 2024; s007lbl.pdf.) is a fixed-dose ophthalmic formulation combining Timolol Maleate 0.5%, a nonselective beta-blocker, with Brimonidine Tartrate 0.2%, a selective alpha-2 adrenergic agonist. Clinical evidence shows that Combigan provides superior intraocular pressure (IOP) reduction compared to monotherapy with either Timolol or Brimonidine alone, (Craven et al., 2005), and it also effectively minimizes IOP fluctuations (Spaeth et al., 2011). Importantly, the combination does not increase the risk of adverse effects beyond those seen with the individual agents, (Larsson, 2001), and has even been linked to a lower incidence of ocular allergy than brimonidine monotherapy (Motolko, 2008). Furthermore, multiple studies have found Combigan to outperform other fixed combinations in reducing IOP, (García-Feijoó et al., 2010), while offering better overall tolerability (Gulkilik et al., 2011).

**FIGURE 1:**
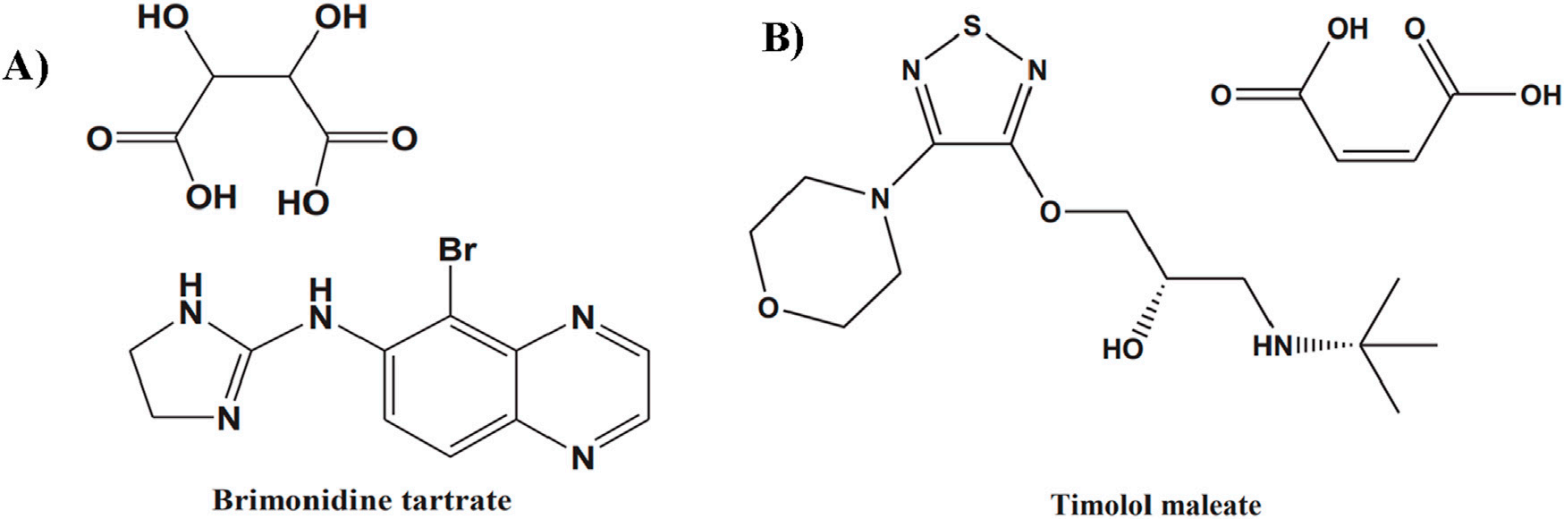
The chemical structure of brimonidine tartrate **(A)** and timolol maleate **(B)**.

Brimonidine Tartrate (BT) is the tartrate salt of brimonidine (5-bromo-6-(2- imidazolin-2-ylamino) quinoxaline D-tartrate), (PubChem, 2010), an imidazole-based compound that acts as a highly selective alpha-2 adrenergic receptor agonist. By engaging this G-protein-coupled receptor, (Mestareehi and Abu-Farsakh, 2024), brimonidine suppresses intracellular adenyl cyclase activity, leading to decreased production of aqueous humor (AH) (Figure 1A) (Dinçel et al., 2023). The drug exhibits a relatively brief systemic elimination half-life (t_1_/_2_) of about 2–3 h. Timolol maleate (TM) is chemically described as 2-propanol, 1-(1,1-dimethylethyl)amino-3-[[4-(4-morpholinyl)-1,2,5-thiadiazol-3-yl]-,(S)-,(Z)-2-butenedioate (1:1) (salt)) (PubChem, 2012). TM is classified as a non-selective β-adrenergic blocker lacking both intrinsic sympathomimetic and membrane-stabilizing properties, as illustrated in Figure 1B (Timolol maleate, 2013). Its therapeutic effect in glaucoma arises from blocking β-adrenergic receptors in the ciliary epithelium, thereby lowering aqueous humor production and reducing intraocular pressure (Larsson, 2001).

The United States Pharmacopeia recommends HPLC methods for analyzing this compound in both tablets and ophthalmic solutions, whereas the British Pharmacopoeia outlines a direct spectrophotometric assay for its determination in the same dosage forms (Walash and El-Shaheny, 2016). Several liquid chromatographic methods have been described for quantifying brimonidine tartrate and timolol maleate in pharmaceutical formulations (Mathrusri Annapurna, 2017; Patel et al., 2023). In a previously reported method, the observed retention times for brimonidine tartrate and timolol maleate were notably brief, measured at approximately 0.51 min and 0.65 min, respectively; (Büker and Dinç, 2017); however, these rapid elution times raise concerns that potential degradants or impurities could be overlooked. Moreover, the method did not include forced degradation stability studies, leaving the influence of possible degradation products on the analytical results unexamined. Another published method relied on an unusually narrow linear concentration range (4–24 μg/mL, 10–60.0 μg/mL) for of brimonidine tartrate and timolol maleate respectively, which is not recommended for method development due to its limited applicability and increased risk of analytical errors (Elshanawane et al., 2011; Mestareehi, 2025a). This restricted range also hampers the method’s ability to evaluate drug stability and detect degradation products effectively. As a result, the absence of a robust stability-indicating capability emerges as a significant limitation, highlighting the need for a more comprehensive and reliable analytical method. While numerous studies have independently examined the chromatographic analysis of Brimonidine Tartrate and Timolol Maleate, there is still a clear gap in the literature regarding the development of a single RP-HPLC method capable of both simultaneously quantifying these compounds and serving as a stability-indicating assay for combination formulations. To address this gap, the present study aims to develop a simple, rapid, precise, and accurate RP-HPLC stability-indicating method specifically for Combigan®. The method will be validated following ICH and FDA guidelines to ensure compliance with regulatory requirements for quality control and stability testing. Ultimately, this method is intended to offer a reliable analytical tool for monitoring the stability of Combigan®, safeguarding its potency, safety, and therapeutic efficacy throughout its shelf life.

## Materials and methodology

### Chemicals and reagents

Brimonidine Tartrate (BT, USP) and Timolol Maleate (TM, USP) were used as active pharmaceutical ingredients. Maleic acid from Sigma-Aldrich ReagentPlus®, ≥99% purity. Hydrochloric acid (12 N) and sodium hydroxide was purchased from EM Science (United States). Acetonitrile (ACN, HPLC grade) was sourced from Fisher Scientific (United States). Potassium phosphate monobasic was obtained from Merck & Co. (Germany). Phosphoric acid (85%) was sourced from J.T. Baker (United States), and glacial acetic acid was obtained from Mallinckrodt Inc. (United States). Hydrogen peroxide solutions (30% and 3%) were purchased from Fisher Scientific (United States). Deionized water was prepared using a Milli-Q water purification system (Millipore, Bedford, MA, United States). pH buffer standards at pH 4.0, 7.0, and 9.0 were all procured from Fisher Scientific (United States).

### Chromatography equipment

An Agilent 1,100 series HPLC system equipped with a G1311A quaternary pump, G1329A autosampler injector, G1365B DAD detector, G1316A Column Thermostat, and G1314A UV detector was utilized for chromatographic analysis. Data acquisition and processing were performed using ChemStation software. Additional laboratory equipment included a Mettler Toledo analytical balance (model AB265-S, Switzerland), a pH meter (model 3,540, UK), and a Bandelin-Sonorex ultrasonic bath (model TK 52, Germany). Precise liquid handling was achieved with an Advantage-Lab variable micropipette (capacity up to 1,000 μL). Chromatographic separation of target analytes was carried out using a Supelco Discovery C18 column (25 cm × 4.6 mm, 5 µm). Prior to use, the mobile phase underwent filtration through a 0.45 μm membrane filter (Millipore, Milford, MA) to ensure clarity and remove particulates. Throughout the method, a Sigma refrigerated centrifuge (Germany) and a Stuart vortex mixer (England) were employed to support sample preparation and processing. Additionally, a UV/VIS spectrophotometer (Hitachi U-2910), and a Thermo Nicolet (IR 200) spectrometer were used during the analysis.

### Pharmaceutical dosage

Combigan® ophthalmic solution, manufactured by Allergan (United States), is formulated to contain Brimonidine Tartrate at a concentration of 0.2% and Timolol Maleate at 0.5%, with NDC 0023-9,211–10.

### Chromatographic conditions

Table 1 summarizes the optimized chromatographic conditions established for the analysis of Brimonidine Tartrate and Timolol Maleate. These carefully selected parameters achieve rapid separation and robust detection, ensuring precise quantification of Combigan® components within a streamlined run time.

**TABLE 1:** Optimized chromatographic conditions for Combigan^®^ method development.

Parameters	Conditions
Column	Supelco Discovery C18, 5 µm (250 × 4.6 mm)
Mobile Phase	20:80 ACN/monobasic potassium phosphate buffer pH 7.0 with 30 mM TEA
RT (retention time)	10.5 ± 0.235 min
Flow Rate	1 mL/min
Sample Injector	15 µL loop
Detection Wavelength	245 nm and 295 nm
Column Temperature	Ambient

### Solution preparation procedures

Comprehensive information on the procedures for sample preparation, the mobile phase formulation, and the analytical techniques employed in this work are described below.

### Mobile phase A (buffer pH 7.0) with 30 mM TEA: To prepare 1 L of 25 mM potassium phosphate monobasic solution at pH 7.0 with 30 mM TEA

Accurately weigh 3.40 g of potassium phosphate monobasic and transfer it into a 1,000 mL beaker. Add 1,000 mL of deionized (DI) water and stir thoroughly until the buffer salts are fully dissolved. add 4.174 mL of TEA. Insert a calibrated pH probe into the solution and adjust the pH to 7.0 as needed. Filter the prepared buffer through a 0.45 µm membrane filter, then sonicate the solution for 20 min to remove any entrapped air bubbles.

### Mobile phase B (100% acetonitrile)

Transfer 1,000 mL of ACN into the mobile phase reservoir and sonicate for 20 min to remove air bubbles.

### Stock solution of brimonidine tartrate (1,000 ppm)

Accurately weigh 50 mg of Brimonidine Tartrate and place it into a 50 mL volumetric flask. Add approximately 40 mL of deionized water and sonicate for about 10 min, or until the compound fully dissolves. Carefully fill the flask to the calibration mark with deionized water and mix well to ensure complete homogenization.

### Stock solution of timolol maleate (1,000 ppm)

Weigh precisely 50 mg of Timolol Maleate and transfer it into a 50 mL volumetric flask. Add around 40 mL of deionized water and sonicate for roughly 10 min or until fully dissolved. Then, top up to the volume mark with deionized water and shake gently to achieve uniform mixing.

### Working standard solutions of BT (200 ppm) and TM (500 ppm)

Transfer 2 mL of Brimonidine Tartrate (1,000 ppm) and 5 mL of Timolol Maleate (1,000 ppm) into a 10 mL volumetric flask. Fill the flask to the 10 mL mark with distilled water and mix thoroughly to ensure a uniform solution.

### Sample combigan preparation (600 ppm of BT& 1,500 ppm of TM)

Transfer 3 mL of Combigan eye drops and transfer it into a 10 mL volumetric flask. Then, fill the flask up to the 10 mL mark with distilled water, mixing thoroughly to ensure uniform dilution.

## Method development and optimization

The main aim of this study was to design a simple, efficient, selective, and accurate RP-HPLC method specifically for quantifying Brimonidine Tartrate and Timolol Maleate (Combigan®) in bulk drug form. A critical goal was also to achieve effective separation of these active ingredients from related impurities and potential degradation products, without requiring extra purification steps. Various experimental parameters were systematically examined to identify optimal chromatographic conditions. Emphasis was placed on obtaining a high number of theoretical plates (reflecting superior column efficiency), sharp and symmetrical peak shapes, reduced tailing in raw material analysis, and robust separation of brimonidine tartrate and timolol maleate from impurities and degradants. Method development included a series of targeted trials to refine and validate these conditions, ultimately ensuring consistent, precise, and reproducible analytical performance (Mestareehi, 2025a).

### Determination of the wavelength of maximum absorbance

**FIGURE 2:**
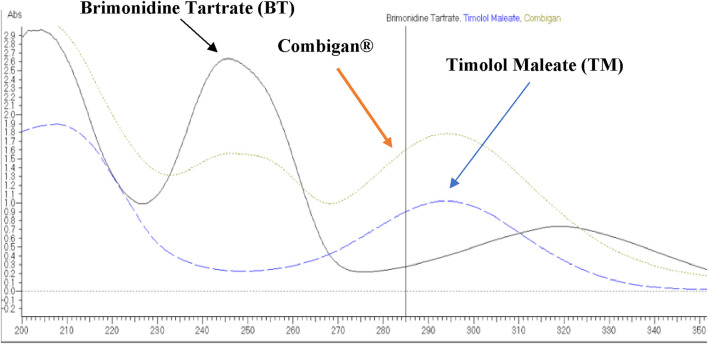
UV Spectrum of Brimonidine Tartrate, Timolol Maleate, and Combigan using a Hitachi UV/VIS Double Beam Spectrophotometer, Model U-2900.

To identify the optimal detection wavelength, standard solutions of brimonidine tartrate (80 ppm), timolol maleate (50 ppm), and Combigan (40/100 ppm, BT/TM) were scanned using UV spectroscopy over the range of 200–400 nm, with the buffer solution serving as the reference blank. Brimonidine Tartrate exhibited maximum absorbance at 245 nm, Timolol Maleate at 295 nm, and Combigan at both 245 nm and 295 nm. Based on these results, 245 and 295 nm were selected as the analytical wavelength for the quantitative determination of Combigan as seen in Figure 2.

### Infrared (IR) study for brimonidine tartrate and timolol maleate

**FIGURE 3:**
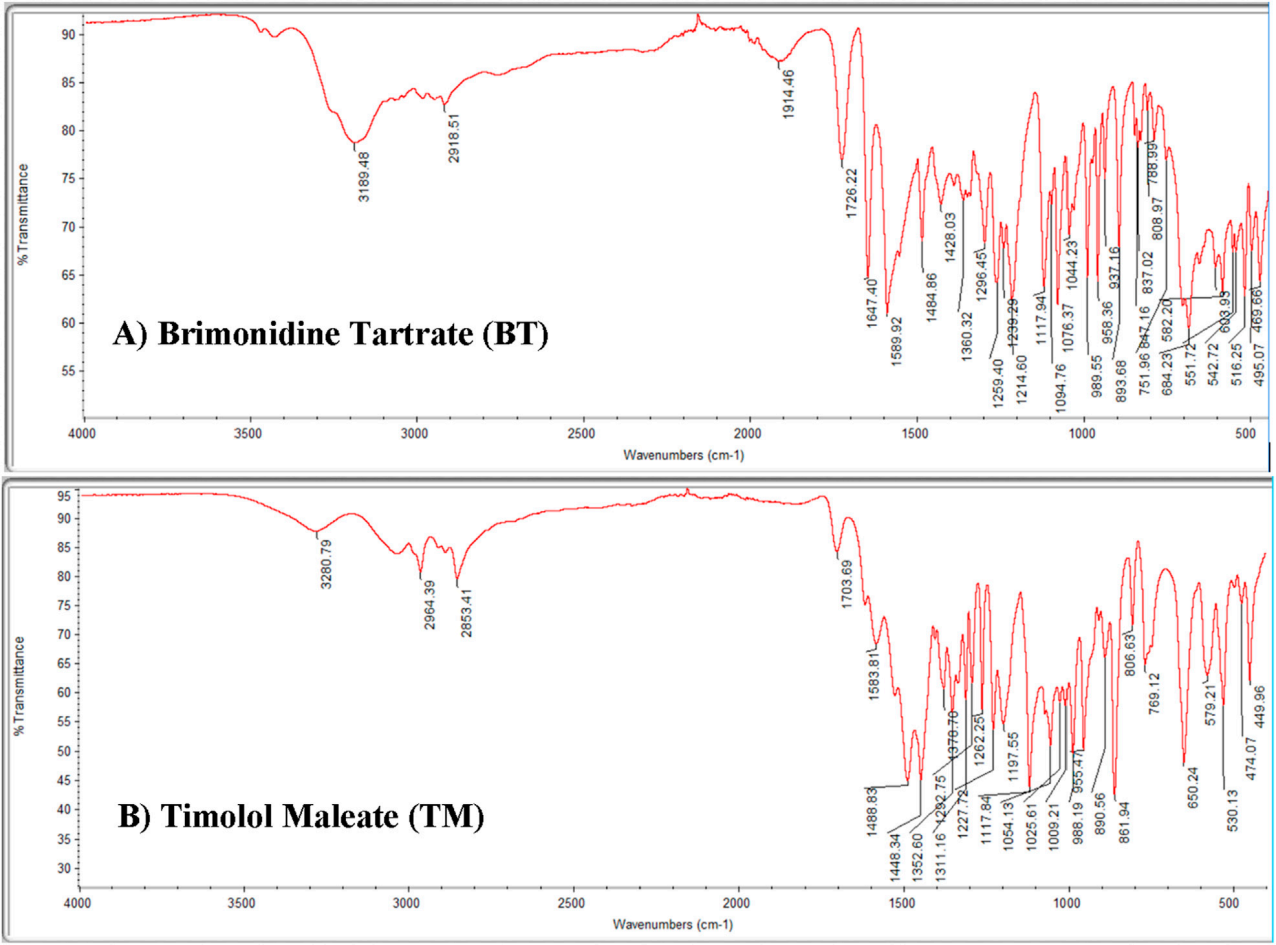
Infrared (IR) spectrum of **(A)** Brimonidine Tartrate and **(B)** Timolol Maleate using Thermo Nicolet IR 200 Spectrometer.

Approximately 100 mg of potassium bromide (KBr) and 2 mg of each sample Brimonidine Tartrate and Timolol Maleate were weighed separately for analysis. Each finely ground drug sample was thoroughly blended with the powdered KBr and then subjected to high pressure to form a transparent pellet. Under pressure, the potassium bromide fuses, embedding the active compound within a stable matrix to produce a thin disk suitable for infrared (IR) analysis. These prepared KBr pellets were carefully placed in the spectrometer’s sample holder for scanning. The resulting infrared spectra, shown in Figure 3, reveal the characteristic absorption bands associated with functional groups present in Brimonidine Tartrate and Timolol Maleate. The identified functional groups are summarized in Table 2.

**TABLE 2:** Key infrared (IR) absorption peaks of brimonidine tartrate, timolol maleate and their functional group assignments.

Observed peak (cm^-1^)	Functional group/Vibration	Assignment
Brimonidine tartrate (BT)		
∼3,198	N–H stretching/O–H stretching	Amidine group in brimonidine and/or O–H from tartrate
∼2,918	Aliphatic C–H stretching	CH_2_ and CH_3_ groups
∼1726	C=O stretching	Carboxylic acid groups in tartrate counterion
∼1,617–1,500	Aromatic C=C stretching	Quinoxaline aromatic ring in brimonidine
∼1,300–1,000	C–N stretching/C–O stretching	Amidine C–N and tartrate C–O groups
∼900–700	Aromatic C–H out-of-plane bending	Substituted aromatic ring
Timolol Maleate (TM)		
∼3,320	N–H stretching/O–H stretching	Secondary amine in timolol and/or hydroxyl group
∼2,984–2,853	Aliphatic C–H stretching	CH_2_ and CH_3_ groups in side chains
∼1703	C=O stretching	Carboxylic acid group in maleate counterion
∼1,639	C=C stretching/N–H bending	Maleate double bond or amine bending
∼1,488–1,444	CH_2_ bending/aromatic C–C stretching	Methylene groups or aromatic ring vibrations
∼1,300–1,000	C–N stretching/C–O stretching	Morpholine ring and other ether or alcohol linkages
∼800–600	Aromatic or alkene C–H out-of-plane bending	Substituted aromatic or alkene hydrogens

### Column selection

**FIGURE 4:**
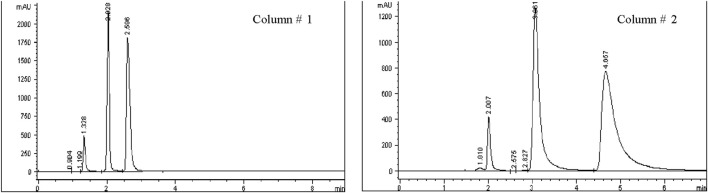
Chromatograms obtained using Columns #1 and #2 (C18, 4.6 × 250 mm, 5 µm) following the injection of Brimonidine Tartrate (200 ppm) and Timolol Maleate (500 ppm).

Five different C18 columns were conditioned by sequential flushing with solvent mixtures of 50:50, 75:25 acetonitrile (ACN): water, and finally 100% ACN, each for 30 min at a flow rate of 1 mL/min. This step ensured compliance with ICH Q2 (R2) guidelines, (Abraham et al., 2010), targeting tailing factors between 0.9 and 2.0 and theoretical plate numbers above 2000 for acceptable peak symmetry and column efficiency. Each column was tested twice, with thorough cleaning and equilibration between runs to avoid cross-contamination. Brimonidine Tartrate and Timolol Maleate solutions (200 ppm and 500 ppm) were injected to evaluate column performance, with the columns maintained at ambient temperature to enhance reproducibility (see Figure 4; Table 3).

**TABLE 3:** Selection of five different C18 columns.

Column	Manufacturer	Type	Part number	Length (mm)	Internal diameter (mm)	Particle size (µm)
1	Agilent Zorbax Rx	C_18_	880967-902	250	4.6	5
2	Agilent Zorbax SB	C_18_	880975-902	250	4.6	5
3	Supelco Discovery	C_18_	45574-03	250	4.6	5
4	Phenomenex Maxsil 5 RP-2	C_18_	006-0391-EO	250	4.6	5
5	Phenomenex Hyperclone BDS	C_18_	00F-4420-EO	150	4.6	5

**FIGURE 5:**
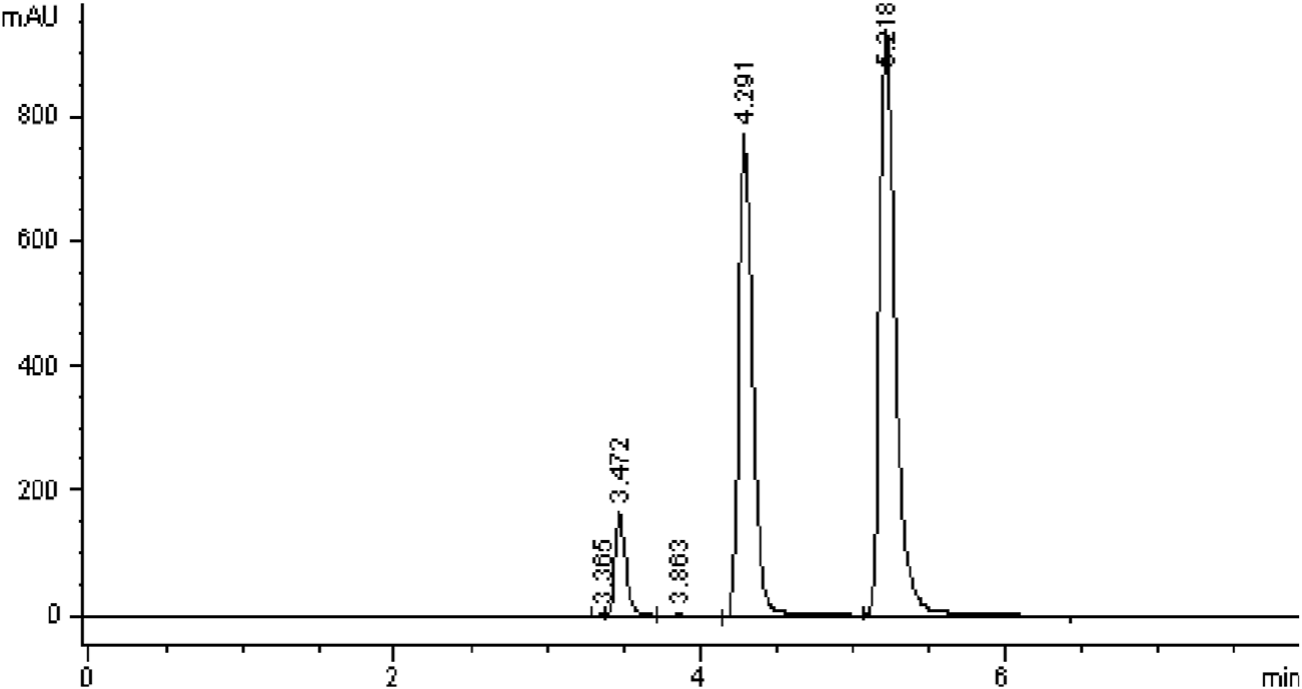
Chromatograms obtained using Column #3 Supelco Discovery (C18, 4.6 × 250 mm, 5 µm) following the injection of Brimonidine Tartrate (200 ppm) and Timolol Maleate (500 ppm).

All five C18 columns were evaluated under identical chromatographic conditions to allow direct comparison. Columns #2, 4, and five failed to meet ICH acceptance criteria due to tailing factors exceeding 2.0, indicating poor peak symmetry (Abraham et al., 2010). Column #1 met the minimum requirements for tailing factor and theoretical plate count but showed lower efficiency compared to Column #3 and had a retention time too short for degradation studies. Ultimately, Column #3 was selected for further method validation, as it provided the best overall performance with a tailing factor ≤2.0 and theoretical plate count ≥2000. Additional details are summarized in Figure 5; Table 4.

**TABLE 4:** Column selection results showing performance data for Peak 1 (Maleic acid), Peak 2 (Brimonidine tartrate), and Peak 3 (Timolol maleate).

Column	Peak	Retention time (min)	Tailing factor	Theoretical plates	Resolution
1	1	1.328	1.603	2,571	-
	2	2.028	1.152	4,928	6.232
	3	2.596	1.754	2,908	3.638
2	1	2.007	1.636	4,469	-
	2	3.061	2.275	2,921	0.639
	3	4.657	3.347	1,277	4.190
3	1	3.472	1.349	12420	-
	2	4.291	1.337	11171	1.226
	3	5.218	1.594	12082	5.368
4	1	2.046	2.603	2,449	-
	2	3.232	1.900	1,471	0.958
	3	4.328	2.743	2050	0.175
5	1	2.019	1.453	7,313	-
	2	3.471	1.916	2,502	0.674
	3	6.560	3.112	882	5.247

**FIGURE 6:**
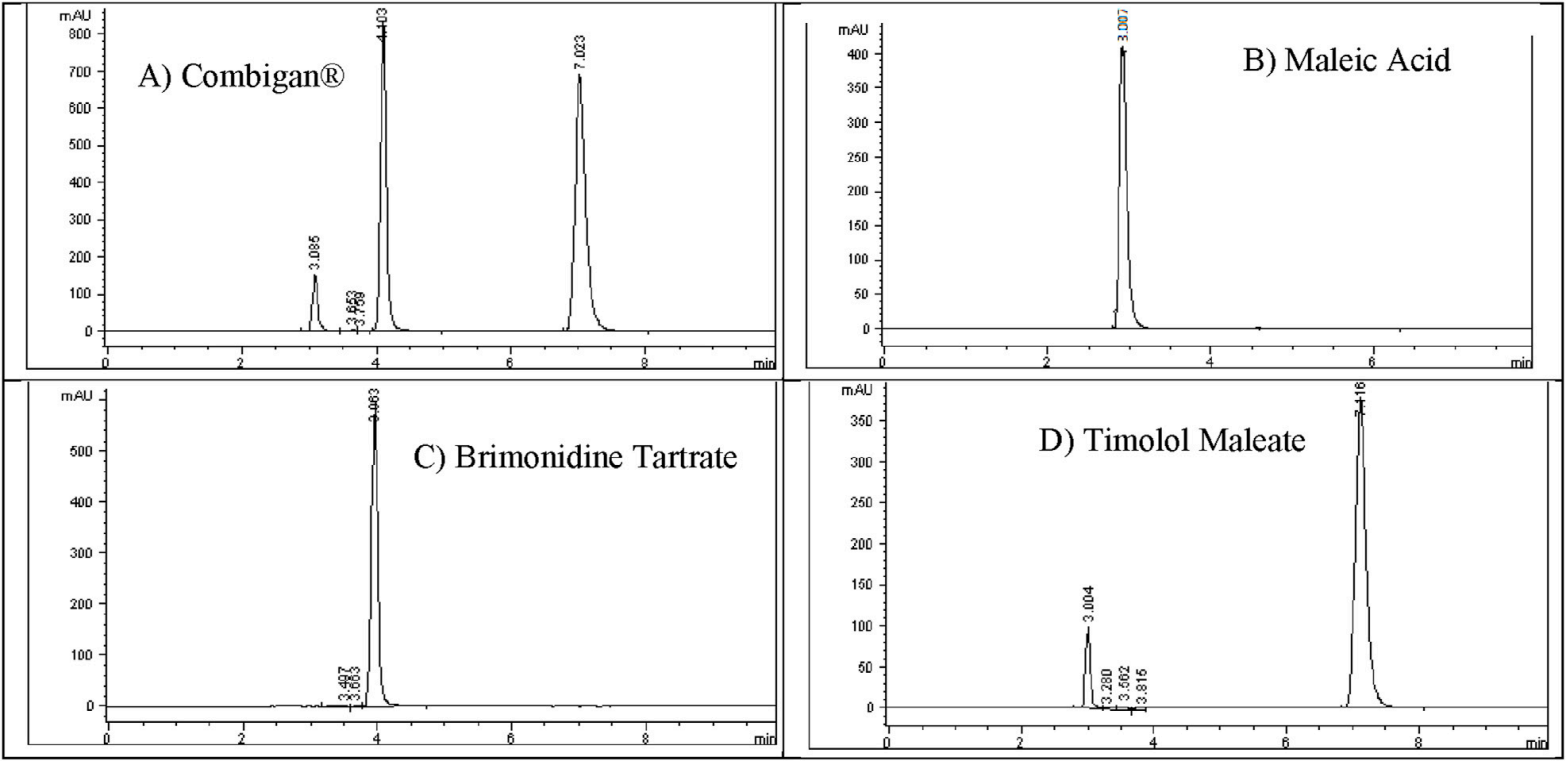
Identification of **(A)** Combigan® peaks by retention time using standard injections of **(B)** maleic acid (100 ppm), **(C)** brimonidine tartrate (200 ppm), and **(D)** timolol maleate (500 ppm).

The Combigan® chromatogram displayed three distinct peaks. To identify them, individual standard solutions of 200 ppm Brimonidine Tartrate, 500 ppm Timolol Maleate, and 100 ppm Maleic Acid were injected under optimized conditions to establish their respective retention times as illustrated in Figure 6.

### Selection pH of the mobile phase

Precise pH control is essential in RP-HPLC to reduce secondary interactions on silica-based columns, which can cause peak tailing and poor separation (Mestareehi, 2025b). Brimonidine Tartrate and Timolol Maleate solutions (200 ppm and 500 ppm) were tested at pH 2.9, 5.0, and 7.0 using a mobile phase of buffer (phase A) and acetonitrile (phase B) in a 80:20 v/v ratio. As summarized in Table 5, pH 7.0 produced the best peak shape and the highest theoretical plate count, indicating superior column efficiency. Therefore, a potassium phosphate monobasic buffer at pH 7.0 was selected for further method development and validation.

**TABLE 5:** Summary of pH Optimization and Selection.

Combigan^®^ ophthalmic solution								
	Brimonidine tartrate				Timolol maleate			
Buffer pH	Retention time (min)	Tailing factor	Theoretical plates	Resolution	Retention time (min)	Tailing factor	Theoretical plates	Resolution
7.0	5.185	1.355	12849	2.164	7.143	1.598	11725	10.057
5.0	4.704	1.181	8,061	1.649	6.452	1.496	12550	7.760
2.9	4.662	0.943	4,864	1.341	6.176	1.643	13171	3.326

### Isocratic elution studies

**FIGURE 7:**
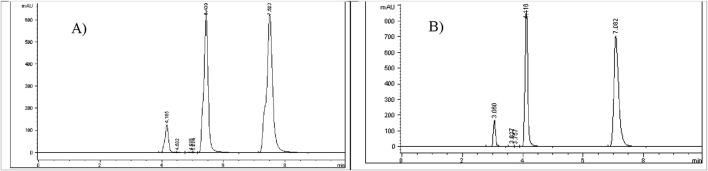
Effect of ACN: buffer pH 7.0 ratios and TEA addition on peak shape and retention of Brimonidine Tartrate and Timolol Maleate. **(A)** *Chromatogram without TEA;* **(B)** *Chromatogram with 30 mM TEA*.

Different ratios of buffer (pH 7.0) and acetonitrile (ACN) were evaluated to achieve retention times around 10 min, allowing adequate separation of impurities and degradants before the main analyte peaks. Brimonidine Tartrate and Timolol Maleate solutions (200 ppm and 500 ppm) were injected under these conditions. As illustrated in Figure 7, initial chromatograms showed peak tailing (TM: Tailing 2.191) due to secondary interactions. To address this, triethylamine (TEA) was added to the buffer, and its concentration was fine-tuned to produce sharper peaks and reduce tailing. Retention times, peak areas, and tailing factors are detailed in Table 6. Further optimization of ACN-to-buffer pH 7.0 ratios containing 30 mM TEA determined that a 20:80 v/v composition was optimal, yielding a retention time near 10 min and improving the resolution of impurities and degradation products.

**TABLE 6:** Summary of retention times, tailing factors, and theoretical plate numbers under different TEA concentrations using a 80:20 buffer pH 7.0: ACN mobile phase.

Combigan^®^ ophthalmic solution						
	Brimonidine tartrate			Timolol maleate		
Solvent strength (%B)	Retention time (min)	Tailing factor	Theoretical plates	Retention time (min)	Tailing factor	Theoretical plates
20	5.646	1.361	10473	9.960	1.972	10702
25	5.185	1.355	12849	7.143	1.598	11725
30	4.856	1.297	7,065	5.894	1.462	9,233

Combigan^®^ ophthalmic solution						
20% B	Brimonidine tartrate			Timolol maleate		
TEA concentration (mM)	Retention time (min)	Tailing factor	Theoretical plates	Retention time (min)	Tailing factor	Theoretical plates
25	5.185	1.355	12849	7.143	1.598	11725
30	4.116	1.109	12497	7.082	1.423	11320
35	4.103	1.092	11234	7.023	1.343	11132

### Nominal concentration selection

**FIGURE 8:**
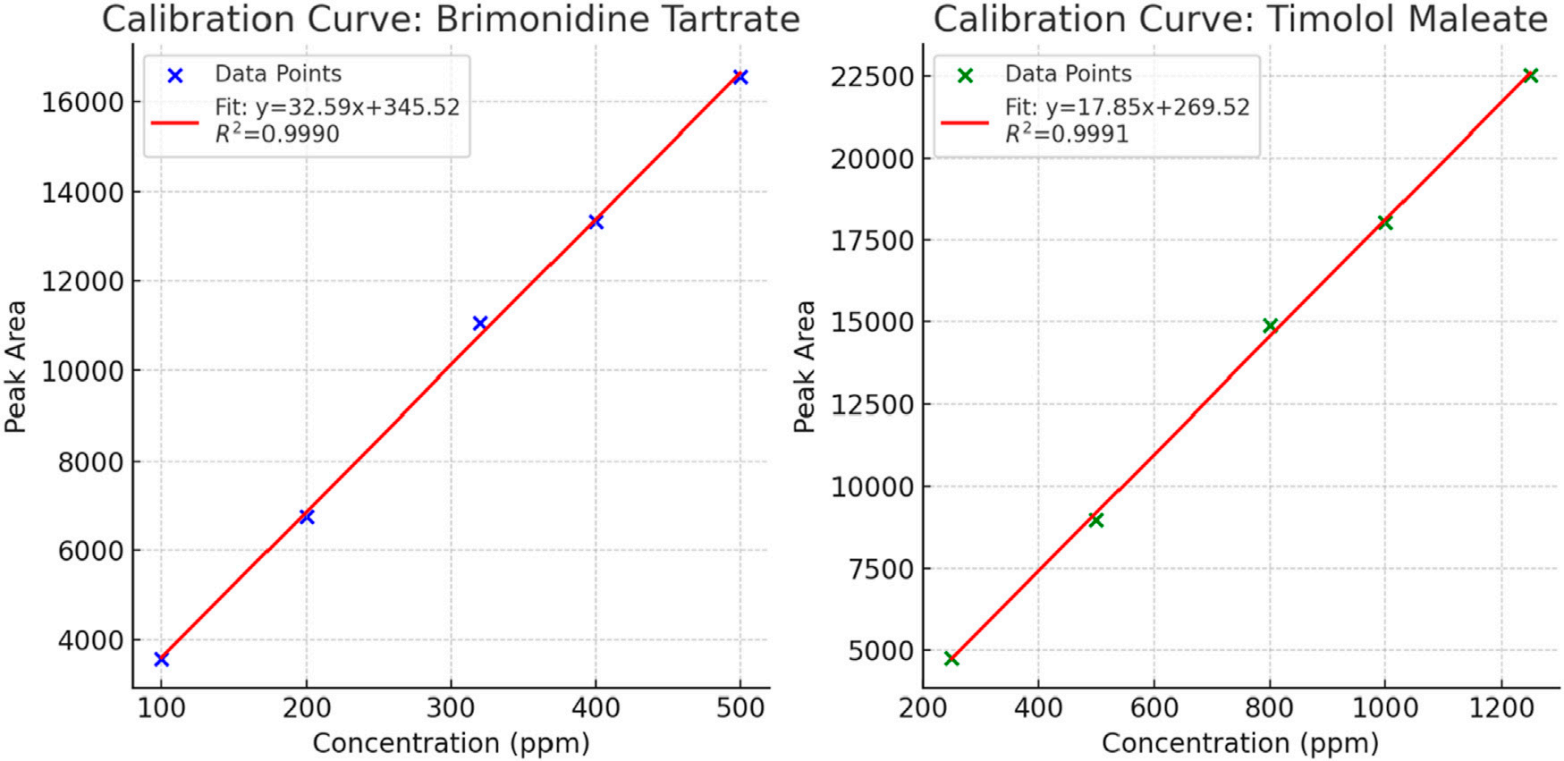
Calibration curves for Brimonidine Tartrate and Timolol Maleate: Peak area *versus* concentration (ppm) with corresponding regression equations and R^2^ values.

To establish nominal concentrations for Brimonidine Tartrate and Timolol Maleate, calibration curves were generated within the detector’s linear range to ensure proportional response and minimize bias. Stock solutions (600 ppm and 1,500 ppm, respectively) were diluted to prepare calibration standards: 100–500 ppm for Brimonidine Tartrate and 250–1,250 ppm for Timolol Maleate. These were analyzed under optimized HPLC conditions, and peak areas (Table 7) showed excellent linearity with R^2^ values of 0.9990 for Brimonidine Tartrate and 0.9991 for Timolol Maleate (Figure 8). As a result, 200 ppm of Brimonidine Tartrate and 500 ppm of Timolol Maleate were chosen as nominal concentrations for further method validation.

**TABLE 7:** Summary of peak areas at different concentrations for determining the nominal concentration.

Brimonidine tartrate		Timolol maleate	
Concentration (ppm)	Peak area	Concentration (ppm)	Peak area
100	3,576	250	4,759
200	6,750	500	8,986
320	11054	800	14890
400	13327	1,000	18043
500	16553	1,250	22514

### Forced degradation studies

Forced degradation studies are a fundamental component in evaluating and understanding the intrinsic stability characteristics of pharmaceutical compounds. This approach systematically subjects the drug to a variety of stringent conditions to explore its degradation pathways and identify potential degradation products. Beyond merely observing degradation, such studies also play a pivotal role in partially validating the specificity of analytical methods developed for detecting related substances, including degradation products (Mestareehi A. H., 2025). Ultimately, this ensures more accurate impurity detection and contributes to safeguarding the quality, efficacy, and safety of pharmaceutical products used in clinical practice.

#### Acid degradation

Transfer 2 mL of the Brimonidine Tartrate stock solution (1,000 ppm) into a screw-cap test tube. Add 2 mL of 3 M HCl, then place the tube on a heating block set to 75 °C and heat for 24 h to induce forced degradation. After heating, allow the solution to return to room temperature, then carefully neutralize by adding 2 mL of 3 M NaOH. Transfer the neutralized solution into a 10 mL volumetric flask and dilute to the mark with deionized water, mixing thoroughly to obtain a final concentration of 200 ppm. In parallel, transfer 2 mL of the Timolol Maleate stock solution (1,500 ppm) into a separate screw-cap test tube. Add 2 mL of 1 M HCl, then heat on a heating block at 75 °C for 24 h. After heating, cool to room temperature and neutralize by adding 2 mL of 1 M NaOH. Dilute the neutralized solution appropriately with deionized water, mixing well to achieve a final concentration of 500 ppm. Before HPLC analysis, confirm that each prepared solution is neutral (pH ∼7) using pH indicator strips. Finally, filter each solution through a 0.45 µm membrane filter to remove particulates, then inject into the HPLC system for analysis.

#### Base (alkali) degradation

Transfer 2 mL of the Brimonidine Tartrate stock solution (1,000 ppm) into a screw-cap test tube. Add 2 mL of 3 M NaOH, then place the tube on a heating block maintained at 75 °C and heat for 24 h to induce forced alkaline degradation. After heating, allow the solution to cool to room temperature and carefully neutralize by adding 2 mL of 3 M HCl. Transfer the neutralized solution to a 10 mL volumetric flask and dilute to volume with deionized water, mixing thoroughly to achieve a final concentration of 200 ppm. In parallel, transfer 2 mL of the Timolol Maleate stock solution (1,500 ppm) into a separate screw-cap test tube. Add 2 mL of 0.05 M NaOH, then heat on a heating block at 75 °C for 24 h to induce degradation. After heating, cool the solution to room temperature and neutralize by adding 2 mL of 0.05 M HCl. Dilute the neutralized solution as needed with deionized water, mixing well to reach a final concentration of 500 ppm. Prior to HPLC analysis, verify that both prepared solutions are at neutral pH (∼7) using pH indicator strips. Finally, filter each solution through a 0.45 µm membrane filter to remove any particulates before injecting into the HPLC system for analysis.

#### Hydrogen peroxide degradation (oxidation)

Transfer 2 mL of the Brimonidine Tartrate stock solution (1,000 ppm) into a screw-cap test tube. Add 2 mL of 3% hydrogen peroxide (H_2_O_2_), then place the tube on a heating block maintained at 75 °C and heat for 24 h to induce oxidative degradation. After cooling the solution to room temperature, transfer it into a 10 mL volumetric flask, dilute to volume with deionized water, and mix thoroughly to obtain a final concentration of 200 ppm. Prior to HPLC analysis, filter the prepared solution through a 0.45 µm membrane filter to remove any particulates. In parallel, transfer 2 mL of the Timolol Maleate stock solution (1,500 ppm) into a separate screw-cap test tube. Add 2 mL of 0.05% hydrogen peroxide (H_2_O_2_) and heat the mixture on a heating block at 75 °C for 24 h. Once cooled to room temperature, transfer the solution int a 6 mL volumetric flask, dilute to the mark with deionized water, and mix thoroughly to achieve a final concentration of 500 ppm. Filter this solution as well through a 0.45 µm membrane filter before injection into the HPLC system.

#### Thermal degradation (heat)

Transfer 2 mL of the Brimonidine Tartrate stock solution (1,000 ppm) into a screw-cap test tube and place it on a heating block set to 75 °C for 24 h to induce thermal degradation. After heating, allow the solution to cool to room temperature. Transfer the entire contents into a 10 mL volumetric flask, dilute to the mark with deionized water, and mix thoroughly to achieve a final concentration of 200 ppm. Prior to HPLC analysis, filter the prepared solution through a 0.45 µm membrane filter to remove any particulates. Similarly, transfer 2 mL of the Timolol Maleate stock solution (1,500 ppm) into a separate screw-cap test tube and heat under the same conditions (75 °C for 24 h). Once cooled to room temperature, transfer the solution into a 6 mL volumetric flask and dilute to volume with deionized water, mixing well to ensure homogeneity before analysis.

#### Photolysis (UV light) stress study

Weigh approximately 20 mg of Brimonidine Tartrate and place it in a cuvette. Expose the sample to ultraviolet (UV) light for 24 h to induce photodegradation. After exposure, accurately transfer 2 mg of the degraded sample into a 10 mL volumetric flask. Add 5 mL of deionized water, then sonicate for 20 min or until the drug is completely dissolved. Dilute to volume with deionized water and mix thoroughly to achieve a final concentration of 200 ppm. Prior to HPLC analysis, filter the solution through a 0.45 µm membrane filter to remove particulates. Similarly, weigh approximately 50 mg of Timolol Maleate and place it in a cuvette. Expose the sample to UV light for 24 h. Following exposure, accurately transfer 5 mg into a 10 mL volumetric flask. Add 5 mL of deionized water and sonicate for 20 min or until fully dissolved. Bring to volume with deionized water and mix thoroughly to achieve a final concentration of 500 ppm. Filter this solution through a 0.45 µm membrane filter before injecting into the HPLC system.

Brimonidine Tartrate demonstrated notable stability under thermal and photolytic conditions, showing no detectable degradation after 24 h at 75 °C or following exposure to UV light at 245 nm for 24 h. Under acidic stress (3 M HCl at 75 °C for 24 h), minimal degradation was observed (0.35%). Alkaline conditions (3 M NaOH at 75 °C for 24 h) produced slightly higher degradation (1.45%), indicating greater susceptibility to basic hydrolysis. Oxidative stress using 3% H_2_O_2_ at 75 °C for 24 h led to moderate degradation (0.798%). Overall, Brimonidine Tartrate was most sensitive to alkaline and oxidative conditions, while remaining highly stable under heat and UV light as illustrated in Table 8.

**TABLE 8:** Overview of Brimonidine Tartrate and Timolol Maleate Degradation Procedures (Thermal, Photolytic, Acidic, Alkaline, and Oxidative); *: Heat it on a heating block at 75 °C for the specified duration.

Stress condition	Time heated	Temperature*	Area	Degradation
Brimonidine tartrate				
Control	-	-	6,052	-
Heat	24 h	75 °C	6,051	0%
Photolysis	24 h	254 nm	6,050	0%
3 M HCl	24 h	75 °C	6,030	0.363%
3 M NaOH	24 h	75 °C	5,965	1.438%
3% H_2_O_2_	24 h	75 °C	6,005	0.776%
Timolol Maleate				
Control	-	-	8,415	-
Heat	24 h	75 °C	8,414	0%
Photolysis	24 h	295 nm	8,415	0%
3 M HCl	24 h	75 °C	6,450	23.35%
1 M HCl	24 h	75 °C	7,950	5.52%
Stress Condition	Time Heated	Temperature*	Area	Degradation
Control	-	-	8,415	-
1 M NaOH	1 h	75 °C	6,402	23.92%
0.1 M NaOH	1 h	75 °C	7,200	14.43%
0.05 M NaOH	1 h	75 °C	7,949	5.54%
Stress Condition	Time Heated	Temperature*	Area	Degradation
Control	-	-	8,415	-
3% H_2_O_2_	1 h	75 °C	3,604	57.17%
0.5% H_2_O_2_	1 h	75 °C	7,610	9.57%

*: The samples were heated on a heating block at 75 °C for the specified duration.

Timolol Maleate demonstrated excellent stability under thermal (24 h at 75 °C) and photolytic (24 h at 295 nm) conditions, showing no detectable degradation. However, it showed significant sensitivity to both acidic and basic hydrolysis, with degradation increasing alongside acid or alkali strength: 22.5% degradation in 3 M HCl and 24.7% in 1 M NaOH. Lower concentrations of acid (1 M HCl) and base (0.05 M NaOH) resulted in 5.8% and 5.6%, respectively. Timolol Maleate was especially susceptible to oxidative stress, with 58.5% degradation after exposure to 3% H_2_O_2_, indicating pronounced sensitivity to oxidation. Overall, the data highlight that Timolol Maleate is chemically stable under heat and light but degrades notably under strong acidic, basic, and oxidative conditions as seen in Table 8.

### Mixed degradation study (acid, base, and oxidation)

**FIGURE 9:**
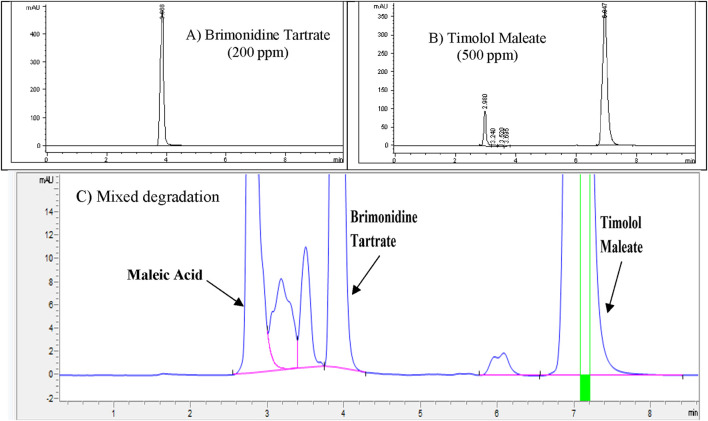
**(A)** Control chromatogram of **(A)** Brimonidine Tartrate (200 ppm) and **(B)** Timolol Maleate (500 ppm) showing baseline retention; **(C)** Zoomed chromatogram after mixed degradation, illustrating complete separation of degradation peaks from main analyte peaks.

The purpose of the mixed degradation study was to evaluate whether the developed HPLC method could effectively separate Brimonidine Tartrate and Timolol Maleate from their potential degradation products and impurities, thus ensuring reliable quantification and purity assessment (Abraham et al., 2010). To establish a baseline, control samples containing 200 ppm of Brimonidine Tartrate, and 500 ppm of Timolol Maleate were prepared and injected into the HPLC system. The resulting chromatogram, presented in Figure 9A, was used to calculate the percentage degradation after subjecting the samples to stress conditions. For the degradation mixture, 1 mL aliquots of Brimonidine Tartrate stress samples that showed less than 10% degradation (treated with 3 M HCl for 24 h, 3 M NaOH for 24 h, and 3% H_2_O_2_ for 24 h) were combined. Similarly, 1 mL aliquots of Timolol Maleate stress samples (treated with 1 M HCl for 24 h, 0.05 M NaOH for 1 h, and 0.5% H_2_O_2_ for 1 h) were mixed. The combined solution was filtered using a 0.45 µm membrane filter and analyzed. The chromatogram from this mixed degradation test is shown in Figure 9B. The analysis demonstrated that all degradation peaks were completely separated from the main Brimonidine Tartrate and Timolol Maleate peaks, which appeared at approximately 5.8 min and 8.8 min, respectively. This clear resolution confirms the method’s specificity and its ability to distinguish the active ingredients from their degradation products. Overall, the findings confirm that the developed RP-HPLC method is stability-indicating and fit for accurate quantification of Brimonidine Tartrate and Timolol Maleate in the presence of degradants. A summary of the degradation results for each stress condition is provided in Table 9.

**TABLE 9:** Summary of degradation results under different stress conditions for Brimonidine Tartrate and Timolol Maleate; *: Heat it on a heating block at 75 °C for the specified duration.

Stress condition	Exposed time	Temperature (°C) *	Color	Peak area	% degradation
Brimonidine tartrate					
None	None	None	Clear	6,052	0%
3M HCl	24 h	75	Clear	6,030	0.363%
3M NaOH	24 h	75	Clear	5,965	1.438%
3.0% H_2_O_2_	24 h	75	Clear	6,005	0.776%
Mixture solution	-	-	Clear	5,960	1.520%
Timolol Maleate					
None	None	None	Clear	8,415	----
1M HCl	24 h	75	Clear	7,950	5.52%
0.05M NaOH	One hour	75	Clear	7,949	5.54%
0.5% H_2_O_2_	One hour	75	Clear	7,610	9.57%
Mixture solution	-	-	Clear	7,595	9.74%

## Method validation

To comply with GLP and GMP standards, the developed analytical method was validated following ICH Q2A/Q2B, (Abraham et al., 2010), FDA, (Beers, 2015), and USP (USP, 2013) guidelines. Key validation parameters included:

- System Suitability: Confirmed consistent HPLC performance before analysis.
- Specificity: Ensured clear separation of BT and TM from impurities and degradants.
- Robustness: Tested stability under minor changes in pH and solvent composition.
- Solution Stability: Verified analyte stability during the test period.
- Linearity and Range: Confirmed accurate detection across relevant concentrations.
- Accuracy and Precision: Assessed repeatability, injection consistency, and analyst variability.
- LOD and LOQ: Defined the lowest detectable and quantifiable levels.

This comprehensive validation demonstrated the method’s reliability and suitability for routine analysis.

### System suitability

System suitability testing (SST) is an essential step in HPLC analysis, as outlined in ICH Q2 (R1) guidelines, ensuring the entire system including equipment, software, and columns is operating consistently and accurately before running actual samples (Abraham et al., 2010). Key acceptance criteria include % RSD of replicate injections not more than 1%, %RSD of retention times not more than 1, number of theoretical plates at least 2000, tailing factor between 0.9 and 2, capacity factor greater than 2, resolution between peaks above 2, and drift within 2%. By meeting these parameters the method confirms it can deliver precise and reproducible results and supports the reliability of subsequent analytical testing (Research, 2020).

System suitability evaluation was carried out by injecting two prepared working standard solutions of Brimonidine Tartrate (200 ppm, labeled as WS #1 and WS #2) and Timolol Maleate (500 ppm, also labeled as WS #1 and WS #2). To verify the consistency and reliability of the HPLC system, WS #1 was injected sequentially six times, while WS #2 was injected twice, as detailed in Table 10. These injections provided data to calculate critical system suitability metrics, such as the %RSD of peak areas and retention times for both WS #1 and WS #2, confirming stable detector response and reproducible retention behavior. Additionally, tailing factors and theoretical plate counts were determined for each analyte peak to assess peak symmetry and column performance, ensuring the method’s robustness and precision.

**TABLE 10:** System suitability results for brimonidine tartrate and timolol maleate working standards.

Standard 1	Retention time	Retention time %RSD	Retention time % Drift	Tailing factor	Theoretical plates	Peak area	Peak area %RSD	Peak area % Drift
Brimonidine Tartrate								
Injection 1	3.963	0.153%	0.029%	0.999	8201	6155	0.847%	0.550%
Injection 2	3.965			0.994	7985	6099		
Injection 3	3.953			0.997	8283	6138		
Injection 4	3.954			0.983	7940	6051		
Injection 5	3.952			0.987	7932	6018		
Injection 6	3.951			0.994	8277	6097		
Standard 2	Retention Time	Retention Time %RSD		Tailing Factor	Theoretical Plates	Peak Area	Peak Area %RSD	
Injection 1	3.959	0.054%		0.990	7961	6097	0.875%	
Injection 2	3.956			0.988	8198	6022		
Timolol Maleate								
Injection 1	6.87	0.163%	0.022%	1.253	8689	8432	0.604%	0.137%
Injection 2	6.869			1.264	8701	8406		
Injection 3	6.845			1.259	8640	8329		
Injection 4	6.848			1.245	8648	8415		
Injection 5	6.848			1.244	8646	8378		
Injection 6	6.853			1.231	8659	8305		
Standard 2	Retention Time	Retention Time %RSD		Tailing Factor	Theoretical Plates	Peak Area	Peak Area %RSD	
Injection 1	6.859	0.041%		1.265	8352	6097	0.875%	

* The percentage drift (% Drift) was calculated by using the following equation:

$$\%\text{ Drift}=\frac{\text{As}-\text{Ac}}{\text{As}}\;x\;100$$

Where, As is the average peak area from six consecutive injections of working standard solution #1, and Ac is the average peak area from two injections of working standard solution #2.

### Specificity

**FIGURE 10:**
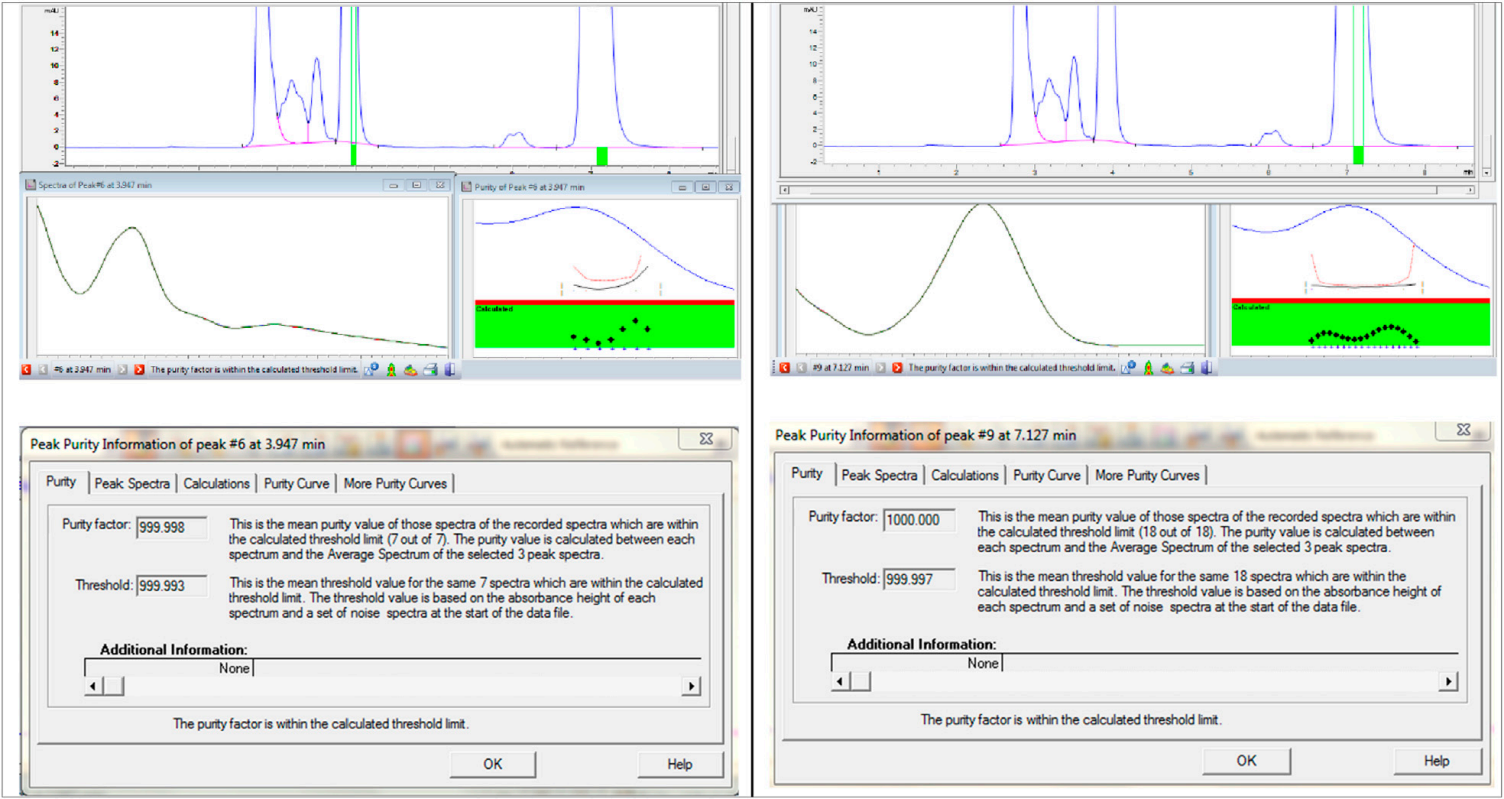
Peak purity information of Brimonidine Tartrate and Timolol Maleate.

Specificity ensures the method can accurately detect and quantify Brimonidine Tartrate and Timolol Maleate without interference from impurities or degradants. Acceptance criteria include a peak purity factor >990, resolution >2 for clear separation, and no overlapping peaks (Mestareehi, 2025a). This confirms the method selectively measures Brimonidine Tartrate and Timolol Maleate in complex sample matrices (Abraham et al., 2010). To prepare the mixed degradation samples, 1 mL portions from acid, base, and oxidative stress solutions each showing less than 10% degradation were combined. For Brimonidine Tartrate, this included 1 mL each from samples treated with 3 M HCl for 24 h, 3 M NaOH for 24 h, and 3% hydrogen peroxide for 24 h. In parallel, 1 mL portions were taken from Timolol Maleate stress solutions exposed to 1 M HCl for 24 h, 0.05 M NaOH for 1 h, and 0.5% hydrogen peroxide for 1 h. The mixed solution was thoroughly blended and passed through a 0.45 µm membrane filter to remove particulates. The prepared sample was then analyzed on an Agilent 1100 HPLC system equipped with a diode array detector (DAD). The chromatographic profile obtained from this mixed degradation study is presented in Figure 10.

### solution stability

The purpose of solution stability testing is to evaluate how the analyte (Brimonidine Tartrate and Timolol Maleate) responds over time when exposed to environmental factors such as temperature, light, and humidity (Research, 2020). This step is essential in method validation to ensure accurate and consistent quantification during routine analysis. Stability was assessed by comparing peak areas from the initial injection with those obtained at 24, 48, and 72 h. Throughout this period, no new peaks or missing peaks were observed in the chromatograms, confirming that the solution remained stable with no significant degradation. The solutions remained stable at room temperature for at least 3 days, as shown in Table 11.

**TABLE 11:** Solution stability of brimonidine tartrate and timolol maleate over 3 Days at room temperature.

Combigan^®^ ophthalmic solution				
	Brimonidine tartrate		Timolol maleate	
Time (hours)	Peak area	% change of peak areas	Peak area	% change of peak areas
0	7,225	-----	9,898	-----
24	7,222	0.042%	9,852	0.465%
48	7,218	0.097%	9,933	0.354%
72	7,215	0.138%	9,924	0.263%

### Method robustness

The robustness of an analytical method evaluates its ability to produce consistent and reliable results despite small, deliberate variations in method parameters. As outlined in ICH guidelines, this step is crucial for confirming that the method can withstand routine fluctuations during practical use (Abraham et al., 2010). In this study, robustness was tested by slightly altering five key parameters: Buffer pH (7.0 ± 0.2), Flow rate (1.0 ± 0.2 mL/min), Detection wavelength (249 ± 2 nm) and (290 ± 2 nm), Mobile phase B composition (20% ± 2% B), and Injection volume (15 ± 2 μL).

The method’s performance under these variations was evaluated using the following acceptance criteria: a tailing factor between 0.9 and 2.0, a theoretical plate count of at least 2000, and resolution (RS) greater than 2.0 between all peaks and the target analyte. Robustness was assessed by deliberately varying critical method parameters: adjusting the mobile phase pH by ± 0.2, changing the percentage of organic solvent (acetonitrile) in the mobile phase by ± 2%, altering the column temperature by ± 2 °C, shifting the detection wavelength by ± 2 nm, and modifying the flow rate by ± 0.2 mL/min. These controlled changes showed no significant impact on chromatographic resolution as shown in Table 12, confirming the method’s robustness.

**TABLE 12:** Method robustness evaluation: Results for mixed degradation samples under parameter variations.

		Brimonidine tartrate		Timolol maleate	
Parameter	Variation	Tailing factor	Theoretical plates	Tailing factor	Theoretical plates
Buffer pH	6.8	0.988	8,983	1.258	9,367
	7.0	1.102	9,658	1.246	9,516
	7.2	0.913	10156	0.902	10230
Wavelength (nm)	293	1.057	9,341	1.223	9,322
	295	1.102	9,658	1.246	9,516
	297	1.009	9,336	1.197	9,321
Wavelength (nm)	243	1.101	7,908	0.9982	8,123
	245	1.090	8,109	1.109	8,201
	247	1.103	8,120	1.009	8,423
% B composition	18% ACN	1.239	9,539	1.265	9,851
	20% ACN	1.102	9,658	1.246	9,516
	22% ACN	0.921	9,620	0.913	9,729
Injection Volume ( μ L)	13	1.209	9,430	1.065	9,457
	15	1.212	9,610	1.246	9,636
	17	1.243	9,543	1.434	9,534
Flow rate mL/min	0.8	1.001	9,399	1.065	9,457
	1	1.120	9,680	1.212	9,636
	1.2	1.321	9,483	1.367	9,534

### Linearity and range for active ingredient

**FIGURE 11:**
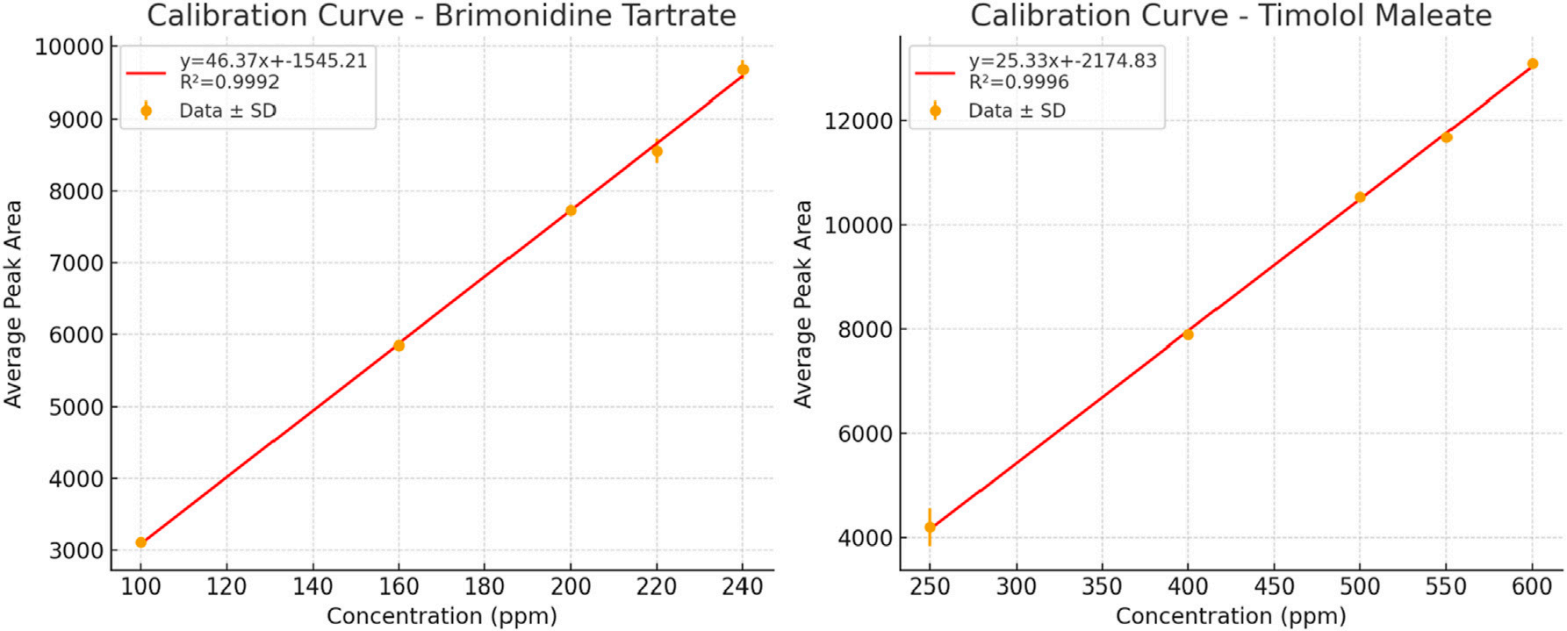
Calibration curve with error bars showing the relationship between peak area and concentration of Brimonidine Tartrate and Timolol Maleate for the linearity study.

The validated linearity of the method was established across the ranges of 0–500 ppm for Brimonidine Tartrate and 250–1,250 ppm for Timolol Maleate using pure standards. For analysis of Combigan® dosage form, additional calibration curves were constructed within narrower ranges (100–240 ppm for Brimonidine Tartrate and 250–600 ppm for Timolol Maleate) to reflect actual sample concentrations. The correlation coefficient (R^2^) for Brimonidine Tartrate and Timolol Maleate must be at least 0.999 to meet acceptance criteria. This ensures a strong linear relationship between concentration and detector response across the tested range, confirming the method’s reliability for accurate quantitative analysis. A stock solution of Brimonidine Tartrate (400 ppm) and Timolol Maleate (1,000 ppm) was used to prepare a series of diluted solutions at concentrations of 100 ppm, 160 ppm, 200 ppm, 220 ppm, and 240 ppm for Brimonidine Tartrate, and 250 ppm, 400 ppm, 500 ppm, 550 ppm, and 600 ppm for Timolol Maleate. These solutions were analyzed using HPLC under optimized isocratic elution conditions. The resulting summary of the peak area results provided in Table 13. The linear regression equations were Y = 46.365 X – 1,545.2 (R^2^ = 0.9992) for Brimonidine Tartrate and Y = 25.334 X – 2,174.8 (R^2^ = 0.9996) for Timolol Maleate, where *Y* is the peak area of the standard solution and *X* is the drug concentration (Figure 11).

**TABLE 13:** Linearity results for Brimonidine Tartrate and Timolol Maleate.

Combigan^®^ ophthalmic solution						
	Brimonidine tartrate			Timolol maleate		
Sample preparation	Concentration (ppm)	Peak area	Average peak area	Concentration (ppm)	Peak area	Average peak area
1	100	3,126	3,117	250	4,649	4,195
2		3,167			3,949	
3		3,058			3,987	
1	160	5,875	5,852	400	7,937	7,891
2		5,902			7,846	
3		5,780			7,890	
1	200	7,753	7,731	500	10597	10539
2		7,651			10567	
3		7,789			10452	
1	220	8,702	8,554	550	11695	11684
2		8,589			11605	
3		8,372			11753	
1	240	9,835	9,676	600	13031	13086
2		9,521			13101	
3		9,672			13126	

### Accuracy

Accuracy of the developed HPLC method focused on evaluating how well the experimentally measured concentrations matched their known theoretical values (Mestareehi, 2025a). Brimonidine Tartrate and Timolol Maleate solutions were prepared in triplicate at three concentration levels: 80%, 100%, and 120% of the nominal concentrations (200 ppm and 500 ppm, respectively). According to the acceptance criteria, the percent recovery for the active ingredients needed to fall within 95%–105% of the target value. Meeting these criteria demonstrates that the method consistently and accurately measures the active ingredients and can also reliably detect impurities and degradants over the tested concentration range. These samples were analyzed by injecting them into the HPLC system, and the resulting peak areas were recorded. The percentage recovery of each active ingredient was then calculated using the linear regression equations obtained from the calibration curves: Y = 46.365 X – 1,545.2 for Brimonidine Tartrate and Y = 25.334 X – 2,174.8 for Timolol Maleate, as shown in Figure 11. By applying these equations, the measured concentrations were compared to the theoretical concentrations to calculate percent recovery. The summarized results in Table 14 confirm that the method meets the established accuracy criteria, achieving recoveries within the acceptable range of 95%–105%.

$$\%\;Recovery=\frac{Csample}{Cstandard}x100$$

**TABLE 14:** Accuracy results for brimonidine tartrate and timolol maleate.

Combigan^®^ ophthalmic solution								
	Brimonidine tartrate				Timolol maleate			
Sample preparation	Concentration (ppm)	Peak area	Average peak area	Recovery	Concentration (ppm)	Peak area	Average peak area	Recovery
1	160	5,764	5,859	99.81%	400	7,757	7,857	99.00%
2		5,843				7,843		
3		5,971				7,971		
1	200	7,598	7,674	99.42%	500	10437	10328	98.71%
2		7,643				10176		
3		7,781				10372		
1	240	9,614	9,558	99.78%	600	12981	13190	101.10%
2		9,535				13253		
3		9,526				13336		

*Csample* is the measured concentration of the sample calculated from the linear regression equation; *Cstandard* is the known (theoretical) concentration of the prepared sample.

### Method precision

Precision in analytical method validation measures how consistently the method produces similar results under defined conditions. As outlined by ICH guidelines, (Abraham et al., 2010), precision is evaluated at three key levels:

1. Repeatability – assesses the closeness of results when the same analyst performs multiple injections or measurements on the same day, using identical equipment and procedures.
2. Intermediate Precision – examines method performance within the same laboratory under varied conditions, such as different analysts, days, or instruments, to identify potential sources of variability.
3. Reproducibility – tests the method’s consistency across different laboratories, ensuring broader reliability of results beyond a single lab setting.

These levels demonstrate the method’s ability to consistently deliver accurate, reliable, and reproducible data under routine and variable conditions.

### Repeatability (method precision)

Repeatability assesses the method’s short-term consistency by examining variability that could result from repeated sample preparation and analysis by the same analyst under identical conditions (Mestareehi et al., 2023). This test highlights the method’s robustness to small operational fluctuations, ensuring reproducible and dependable results in routine analysis. Regulatory guidelines set strict acceptance criteria for repeatability: the % Relative Standard Deviation (%RSD) of peak areas for the target compound must be not more than 1% (NMT 1%) (Purdie, 2021).

In this study, repeatability was evaluated by preparing and injecting six independent samples of Brimonidine Tartrate (200 ppm) and Timolol Maleate (500 ppm) into the HPLC system under the validated chromatographic conditions. The %RSD of the peak areas from these six injections was then calculated, demonstrating the method’s precision and compliance with the defined criteria, as summarized in Table 15.

**TABLE 15:** Repeatability precision results for brimonidine tartrate and timolol maleate.

Combigan^®^ ophthalmic solution								
	Brimonidine tartrate				Timolol maleate			
Sample preparation	Peak area	Average	Standard deviation	Peak area %RSD	Peak area	Average	Standard deviation	Peak area %RSD
1	7,269	7,245	39.80	0.549%	10060	10062	85.28	0.848%
2	7,187				9,945			
3	7,232				10040			
4	7,217				10019			
5	7,295				10194			
6	7,268				10116			

### Injection precision

Injection precision evaluates the method’s ability to consistently produce reliable results by examining instrument-related variability, including potential fluctuations from the injector, detector, column, and data integration process during repeated sample injections (Abraham et al., 2010; Mestareehi et al., 2021). According to the established acceptance criteria, the % Relative Standard Deviation (%RSD) of the peak areas for Brimonidine Tartrate (200 ppm) and Timolol Maleate (500 ppm) must not exceed 1%, ensuring high reproducibility. To assess this, a single preparation of Brimonidine Tartrate (200 ppm) and Timolol Maleate (500 ppm) was injected six consecutive times under the method’s optimized HPLC conditions. The calculated %RSD values confirmed compliance with the acceptance threshold, demonstrating excellent injection precision. The summarized results are provided in Table 16.

**TABLE 16:** Injection precision results for brimonidine tartrate and timolol maleate.

Combigan^®^ ophthalmic solution								
	Brimonidine tartrate				Timolol maleate			
Injection	Peak area	Average	Standard deviation	Peak area %RSD	Peak area	Average	Standard deviation	Peak area %RSD
1	7,468	7,420	63.77	0.859%	10316	10406	84.10	0.808%
2	7,306				10456			
3	7,401				10294			
4	7,413				10431			
5	7,452				10512			
6	7,480				10425			

### Intermediate precision (inter-day precision/ruggedness)

Intermediate precision of the developed analytical method for Brimonidine Tartrate and Timolol Maleate was evaluated to verify its robustness when subjected to deliberate variations, such as different analysts, instruments, columns, and testing days (Abraham et al., 2010; Research, 2020). This level of validation is essential to demonstrate the method’s reliability beyond controlled laboratory conditions. As specified by acceptance criteria, the % Relative Standard Deviation (% RSD) of peak areas for Brimonidine Tartrate at 200 ppm and Timolol Maleate at 500 ppm must not exceed 1.5%. To assess this, six replicate samples of each analyte were prepared and analyzed under the same optimized chromatographic conditions, but on separate HPLC systems, by different analysts, and across multiple days. The resulting data, summarized in Table 17, demonstrate the method’s ability to produce consistent and reproducible measurements despite normal variations in testing conditions. Intermediate precision parameters evaluated for this method include:

- HPLC: 1,100 Series HPLC system with MWD (UV/VIS Detector), Agilent Technologies
- Column: Water XTERRA RP-18 (4.6 × 250 mm, 5 µm)
- Mobile Phase: Solvent A: 25 mM potassium phosphate monobasic buffer, pH 7.0
- Solvent B: 100% ACN.
- Solvent Strength: (80:20 v/v) Buffer pH 7.0: ACN
- Absorbance: 245 nm and 295 nm
- Flow Rate: 1.0 mL/min
- Injection Volume: 15 µL
- Column Temperature: Ambient

**TABLE 17:** Intermediate precision results under varying analysts, instruments, and columns.

Combigan^®^ ophthalmic solution								
	Brimonidine tartrate				Timolol maleate			
Sample preparation	Peak area	Average	Standard deviation	Peak area %RSD	Peak area	Average	Standard deviation	Peak area %RSD
1	7,159	7,239	63.96	0.884%	10155	10065	136.24	1.354%
2	7,243				9,942			
3	7,215				10170			
4	7,319				9,973			
5	7,191				10232			
6	7,308				9,916			

### Limit of detection (LOD) and limit of quantitation (LOQ)

The limit of detection (LOD) was evaluated to determine the lowest concentration at which the analyte could be consistently detected, though not necessarily quantified with precision. Brimonidine Tartrate and Timolol Maleate were used as representative compounds to model impurities and degradation products. The LOD was established by assessing the signal-to-noise (S/N) ratio in the chromatographic response, with an acceptance criterion of an S/N ratio ≥3, indicating the method’s reliable detection capability. To determine the limit of quantitation (LOQ) for Brimonidine Tartrate and Timolol Maleate, a series of dilutions were prepared, and their signal-to-noise ratios were thoroughly evaluated. Preliminary LOQ values were estimated at 0.24 ppm for Brimonidine Tartrate and 0.60 ppm for Timolol Maleate, based on achieving an S/N ratio of ≥10 and a %RSD ≤10%, as summarized in Table 18. To confirm these estimates, fresh solutions at 0.24 ppm and 0.60 ppm were injected ten consecutive times into the HPLC system. This allowed for assessment of both precision and detection reliability. As shown in Table 19, the %RSD for both analytes remained within acceptable limits (≤10%), validating these concentrations as the LOQ for the developed method.

**TABLE 18:** Results of Limit of Detection (LOD) Study for Brimonidine Tartrate and Timolol Maleate solutions.

Combigan^®^ ophthalmic solution			
Brimonidine tartrate		Timolol maleate	
Concentration (ppm)	Signal to noise ratio	Concentration (ppm)	Signal to noise ratio
0.04	1.3	0.10	1.2
0.08	3.5	0.20	3.8
0.12	5.9	0.30	5.2
0.16	7.5	0.40	6.9
0.20	8.3	0.50	7.5
0.24	10.5	0.60	10.1
0.28	11.3	0.70	11.0
0.32	14.4	0.80	13.7
0.36	17.0	0.90	15.8
0.40	18.7	1.00	16.9

**TABLE 19:** Results of Limit of Quantitation (LOQ) Study for Brimonidine Tartrate and Timolol Maleate solutions.

Combigan^®^ ophthalmic solution						
No	Brimonidine tartrate			Timolol maleate		
Injections	Concentration (ppm)	Peak area	Peak area %RSD	Concentration (ppm)	Peak area	Peak area %RSD
1	0.24	9.75	4.709%	0.60	12.88	3.660%
2	0.24	9.02		0.60	13.54	
3	0.24	10.23		0.60	13.72	
4	0.24	9.45		0.60	12.63	
5	0.24	10.07		0.60	12.55	
6	0.24	9.38		0.60	13.09	

## Results and discussion

Glaucoma refers to a group of eye diseases marked by the progressive narrowing of the visual field, typically accompanied by optic nerve damage and characteristic optic disc cupping (Parihar, 2016). Elevated intraocular pressure (IOP) is widely acknowledged as a major risk factor contributing to disease onset and progression (Spaeth et al., 2011). Clinically, glaucoma most commonly presents as either primary open-angle glaucoma or primary angle-closure glaucoma. Its management involves the use of various pharmacological classes designed to reduce IOP through different physiological mechanisms, thereby helping to slow disease advancement and preserve vision (Bengtsson et al., 2007). Typically, first-line treatment involves typical beta-adrenergic blockers or prostaglandin analogues. When further pressure reduction is necessary, these agents can be combined with or supplemented by other therapies, such as miotics, carbonic anhydrase inhibitors, or sympathomimetic drugs (Leske et al., 2004).

Combigan® (Brimonidine Tartrate and Timolol Maleate ophthalmic solution) is specifically indicated for lowering IOP in patients with chronic open-angle glaucoma or ocular hypertension who do not achieve adequate pressure control with single-agent therapy and when the use of COMBIGAN is considered appropriate (García-Feijoó et al., 2010). Beyond reducing IOP, COMBIGAN also helps to minimize long-term fluctuations in eye pressure. By lowering both the average IOP and its variability over time, COMBIGAN aims to slow the progression of visual field loss associated with glaucoma (Bengtsson et al., 2007).

Multiple liquid chromatographic methods have been proposed to quantify Brimonidine Tartrate and Timolol Maleate in pharmaceutical formulations. Some methods report extremely short retention times (around 0.5–0.6 min), (Büker and Dinç, 2017), which risks missing impurities or degradants, especially since forced degradation studies were not performed. Alternative analytical methods have employed relatively limited linear concentration ranges specifically, 4–24 μg/mL for Brimonidine Tartrate and 10–60 μg/mL for Timolol Maleate which restrict their broader applicability and reduce their reliability when used for comprehensive stability studies (Elshanawane et al., 2011). Although several researchers have independently studied the chromatographic analysis of Brimonidine Tartrate and Timolol Maleate, there remains a research gap in developing a unified RP-HPLC method that can simultaneously quantify both drugs and function as a stability-indicating assay in combination dosage forms. This underscores the importance of developing a more comprehensive, robust, and reliable analytical approach to achieve precise quality control and thorough stability evaluation of these ophthalmic formulations.

The system suitability test results show fully resolved peaks with tailing factors near 1, indicating excellent peak symmetry. Over 9,000 theoretical plates were observed, confirming strong column efficiency. For Brimonidine Tartrate, %RSD values for peak area and retention time from six replicates of working standard solution #1 were 0.153% and 0.847%, while for solution #2 (two replicates), they were 0.045% and 0.875%. Drift values were also minimal: % RT Drift at 0.029% and %PA Drift at 0.550%. Similarly, Timolol Maleate exhibited %RSD values of 0.163% and 0.604% (solution #1) and 0.041% and 0.875% (solution #2), with % RT Drift of 0.022% and %PA Drift of 0.137%. These low %RSDs and drift values confirm system repeatability, precision, and stability, satisfying ICH system suitability criteria (Abraham et al., 2010). This test was performed before further analyses to validate system readiness.

The forced degradation study revealed that Brimonidine Tartrate exhibits high stability under thermal and photolytic stress, with no detectable degradation observed after 24 h at 75 °C or under UV irradiation at 245 nm. Under acidic conditions (3 M HCl), the compound showed minimal degradation (0.363%), while exposure to alkaline conditions (3 M NaOH) led to slightly higher degradation (1.438%), suggesting a greater susceptibility to base-catalyzed hydrolysis. Oxidative stress induced by 3% hydrogen peroxide resulted in moderate degradation (0.776%), indicating some vulnerability to oxidation. Overall, these findings confirm that Brimonidine Tartrate maintains robust stability under most stress conditions, with acidic, alkaline and oxidative environments posing the greatest risk for degradation. Similarly, the forced degradation study demonstrated that Timolol Maleate remains chemically stable under thermal stress at 75 °C and photolytic exposure at 295 nm for 24 h, with no measurable degradation observed. However, the drug displayed pronounced sensitivity to hydrolytic conditions, with degradation increasing in proportion to acid and base concentration; degradation reached 23.35% in 3 M HCl and 23.92% in 1 M NaOH. Timolol Maleate was especially susceptible to oxidative degradation, showing substantial degradation of 57.17% when treated with 3% hydrogen peroxide. Lower concentrations and shorter exposure times led to reduced degradation: 5.52% in 1 M HCl (24 h), 5.54% in 0.05 M NaOH (1 h), and 9.57% in 0.5% H_2_O_2_ (1 h). These results highlight that while Timolol Maleate is stable under heat and light, it undergoes significant degradation under strong hydrolytic and oxidative stress, underscoring the need for careful control of these conditions during manufacturing, storage, and formulation to ensure product stability and quality.

The chromatographic analysis successfully detected and resolved the peaks corresponding to Brimonidine Tartrate, Timolol Maleate, and Maleic Acid. All peaks exhibited excellent resolution, thereby fulfilling the specificity criteria set for analytical separation. Additionally, the peak purity index for each analyte exceeded the acceptable threshold, confirming the absence of co-eluting impurities or interference. The specificity assessment demonstrated that the developed method effectively isolates and quantifies these three components even in the presence of degradation products and potential impurities. Peak purity factor (≥999.99), RS ≥ 2.0, and a corresponding three-dimensional chromatogram, which highlight the method’s robustness and demonstrate its adherence to ICH and FDA validation requirements (Abraham et al., 2010).

The results of the robustness study, all parameters complied with ICH guidelines. The tailing factors fell within the acceptable range (0.9–2) and the number of theoretical plates ≥2000, indicating satisfactory column efficiency. Furthermore, the method demonstrated robustness by maintaining consistent performance despite intentional minor variations in solvent composition, buffer pH, flow rate, detection wavelength, and injection volume.

To assess solution stability, a freshly prepared ophthalmic solution containing Brimonidine Tartrate (200 ppm) and Timolol Maleate (500 ppm) was immediately analyzed using the HPLC system. The same solution was then reinjected after 24, 48, and 72 h. Throughout this period, the chromatographic profiles showed neither the appearance of new peaks nor the disappearance of existing ones, indicating that the solution remained stable and did not undergo significant degradation during the study. Specifically, the percent peak area changes for Brimonidine Tartrate at 24, 48, and 72 h was 0.042%, 0.097%, and 0.138%, respectively, while for Timolol Maleate, the percent peak area change over the same time points was 0.465%, 0.354%, and 0.263%.

To evaluate the method’s linearity, five different concentrations of Brimonidine Tartrate and Timolol Maleate standard solutions were prepared, and each was individually injected into the HPLC system. The corresponding peak areas were used to construct calibration curves plotting peak area *versus* concentration. The resulting linear regression analysis yielded correlation coefficients (R^2^) of 0.9992 for Brimonidine Tartrate and 0.9996 for Timolol Maleate. These high correlation coefficients satisfy the acceptance criteria for linearity, confirming that the HPLC method provides a consistent and proportional response across the tested concentration range. Overall, these results demonstrate the method’s validity for precise quantitative determination of the active pharmaceutical ingredients.

The accuracy of the developed RP-HPLC method for quantifying Brimonidine Tartrate and Timolol Maleate in Combigan® ophthalmic solution was evaluated by recovery studies at three concentration levels corresponding to 80%, 100%, and 120% of the target assay concentrations. Each level was analyzed in triplicate. For Brimonidine Tartrate, the average recovery values obtained were 99.81% at 160 ppm, 99.42% at 200 ppm, and 99.78% at 240 ppm. Similarly, for Timolol Maleate, the recoveries were 99.00% at 400 ppm, 98.71% at 500 ppm, and 101.10% at 600 ppm. All recoveries fell well within the generally accepted range of 95%–105%, demonstrating excellent method accuracy across the studied concentration range. These results confirm that the method provides accurate quantification without interference from excipients or other components present in the formulation. The consistency of recoveries across different concentration levels also supports the robustness and reliability of the method for routine quality control and stability testing of Combigan® ophthalmic solution.

To further evaluate the accuracy and precision of the proposed method, results were statistically compared with those of reported methods using the Student’s t-test and F-test at the 95% confidence level (Table 20). The proposed analytical method demonstrated high accuracy and precision for the determination of Brimonidine Tartrate and Timolol Maleate in comparison with the reference method (Elshanawane et al., 2011). Statistical evaluation using the F-test revealed that the proposed method exhibited significantly lower variance (Brimonidine Tartrate: F = 206,611; Timolol Maleate: F = 7,963), indicating superior precision. The t-test results (Brimonidine Tartrate: t = −2.04; Timolol Maleate: t = −2.43) showed no significant difference between the means of the proposed and reference methods at the 95% confidence level, confirming the reliability and suitability of the proposed method for routine analysis. Overall, these findings demonstrate that the proposed method is statistically comparable to the reference method while offering enhanced precision.

**TABLE 20:** Statistical comparison of the proposed and reference methods for Brimonidine Tartrate and Timolol Maleate. Data are mean ± SD. F and t values with their 95% confidence critical limits (F_crit, t_crit) are shown. F-test evaluates precision; t-test evaluates accuracy.

Drug name	Recovery ±SD		Calculated t- values	t_critical (α = 0.05)	Calculated F- values	F_critical (α = 0.05)
	Proposed methods	References method				
Brimonidine Tartrate	99.67 ± 0.0022	100.85 ± 1.00	206,611	2.776	−2.04	19
Timolol Maleate	99.60 ± 0.013	101.41 ± 0.95	7,963	2.776	−2.43	19

To evaluate repeatability, six individually prepared samples of Brimonidine Tartrate (200 ppm) and Timolol Maleate (500 ppm) were analyzed under optimized HPLC conditions. Precision was assessed by calculating the percent relative standard deviation (%RSD) of the peak areas. The %RSD values for Brimonidine Tartrate and Timolol Maleate were 0.549% and 0.848%, respectively. According to ICH guidelines, a %RSD not exceeding 2.0% is considered acceptable, and these results demonstrate that the method consistently delivers precise measurements for both analytes under the same conditions. Injection precision was further evaluated by injecting a single sample of Brimonidine Tartrate and Timolol Maleate six consecutive times into the HPLC system. The resulting %RSD values, 0.859% for Brimonidine Tartrate and 0.808% for Timolol Maleate, also fell within the acceptable range, confirming the method’s reliability for repeated injections. Additionally, intermediate precision was assessed by preparing six separate samples and analyzing them on a different HPLC system. The %RSD values obtained, 0.884% for Brimonidine Tartrate and 1.354% for Timolol Maleate, complied with the specified acceptance criteria. Table 21 presents the results from both analysts, allowing proper calculation of inter-day precision and clear presentation of the data. Overall, these results confirm that the method maintains precision and robustness across repeatability, injection precision, and intermediate precision assessments, meeting the requirements set out in the validation protocol.

**TABLE 21:** The data from both analysts (1; Repeatability Precision 2; Intermediate Precision) for Combigan^®^ ophthalmic solution.

Combigan^®^ ophthalmic solution						
	Brimonidine tartrate			Timolol maleate		
Analyst	Average peak area	Standard deviation	Peak area %RSD	Average peak area	Standard deviation	Peak area %RSD
1	7,239	63.96	0.884%	10155	136.24	1.354%
2	7,245	39.80	0.549%	10062	85.28	0.848%

The limit of detection (LOD) for Brimonidine Tartrate and Timolol Maleate was established by preparing a series of dilutions from the stock solution. Each prepared dilution was examined on the HPLC system, and the corresponding signal-to-noise ratios were determined based on the generated chromatograms. The concentrations of 0.08 ppm for Brimonidine Tartrate and 0.20 ppm for Timolol Maleate produced signal-to-noise ratios of 3.5 and 3.8, respectively. These values satisfy the acceptance criterion of a signal-to-noise ratio ≥3, and thus were designated as the LODs for the method. To establish the limit of quantitation (LOQ), test solutions at concentrations of 0.24 ppm for Brimonidine Tartrate and 0.60 ppm for Timolol Maleate were each injected into the HPLC system in ten replicate runs. Analysis of the resulting chromatograms showed %RSD values for peak areas of 4.70% and 3.66%, both well below the LOQ acceptance threshold of ≤10%. These findings demonstrate that the developed method is sufficiently precise to quantify both analytes reliably at these low concentrations, meeting validation requirements for LOQ performance.

To further verify the Limit of Detection (LOD) and Limit of Quantitation (LOQ) values, (Mestareehi, 2025b), the International Council for Harmonisation (ICH) recommends several calculation methods depending on the analytical technique, analyte characteristics, and method suitability (Abraham et al., 2010). One widely accepted method involves using the standard deviation of the response (σ) and the slope of the calibration curve (S), calculated with the formulas:

$$LOD=3.3X\left(\frac{\sigma }{S}\right)\text{ and }LOQ=10X\left(\frac{\sigma }{S}\right)$$

Using data from the linearity study, we determined σ and S for both Brimonidine Tartrate and Timolol Maleate. Applying these formulas, the calculated LOD and LOQ for Brimonidine Tartrate were 0.099 ppm and 0.30 ppm, respectively, which correspond closely to our experimentally obtained signal-to-noise results (LOD = 0.08 ppm, LOQ = 0.24 ppm). Similarly, for Timolol Maleate, the calculated LOD and LOQ were 0.218 ppm and 0.66 ppm, aligning well with the signal-to-noise based findings (LOD = 0.20 ppm, LOQ = 0.60 ppm). These results confirm that our method validation complies with ICH and USP guidelines, supporting the reliability of the developed analytical method.

In summary, this validated RP-HPLC method addresses significant analytical limitations reported in previous studies by offering a broader linearity range, robust stability-indicating performance, and compliance with international validation standards. Consequently, this method offers a scientifically robust and practically applicable approach for thorough quality control and stability evaluation of combination ophthalmic formulations, thereby reinforcing ongoing initiatives to preserve therapeutic effectiveness and ensure patient safety in the treatment of glaucoma.

To evaluate the environmental sustainability of the developed method, three complementary green analytical chemistry (GAC) metrics were applied: the Analytical Eco-Scale, the Green Analytical Procedure Index (GAPI), and the AGREE evaluation (Yin et al., 2024), (Kaya et al., 2024). The Eco-Scale was calculated by assigning penalty points for hazardous reagents, waste generation, and energy consumption. The main contributor to penalty points was the use of acetonitrile (ACN) as organic solvent in the mobile phase and the direct disposal of waste. Considering dilution as the only sample preparation, room temperature operation (25 °C), and a short chromatographic run time (10 min), the method achieved an Eco-Scale score of ∼75, which classifies it as an acceptable green method (scores ≥75). Moreover, the Green Analytical Procedure Index (GAPI) tool was used to obtain a holistic assessment covering the entire analytical workflow from sample collection to final determination. The pictogram for this method showed a mixture of green and yellow zones, reflecting the environmentally favorable aspects such as simple dilution, absence of derivatization, and low energy consumption. However, red zones were observed in solvent usage and waste management, as ACN is toxic, and waste was directly discarded without treatment. Importantly The AGREE evaluation, based on the 12 principles of green analytical chemistry, generated a circular pictogram and a numerical score. The method scored 0.57 (out of 1.0), indicating moderate greenness. High scores were obtained for minimal sample preparation, short analysis time, and absence of derivatization. Lower scores were assigned to principles related to renewable solvents, waste management, and operator safety, due to the use of ACN and its direct disposal. Overall, the three evaluation tools confirmed that the proposed RP-HPLC method possesses several environmentally favorable features such as simplicity, short analysis time, and low energy consumption. However, the main limitations were related to solvent selection and waste handling. Future improvements could include replacing ACN with a greener alternative (e.g., ethanol or water-rich mobile phases), implementing solvent-recycling strategies, and adopting proper hazardous waste management practices.

## Conclusion

This study successfully developed and validated a robust, accurate, and precise RP-HPLC method for the simultaneous quantification of Brimonidine Tartrate and Timolol Maleate in Combigan® ophthalmic solution. Addressing gaps found in previously published methods, such as insufficient linear ranges, inadequate stability testing, and lack of a unified assay. The new method demonstrated excellent linearity across the tested concentration ranges (R^2^ > 0.999), strong system suitability with high theoretical plate counts and low tailing factors, and compliance with ICH acceptance criteria in all validation parameters. The method proved highly repeatable and precise, as confirmed by low %RSD values in repeatability, injection precision, and intermediate precision assessments. Robustness testing further showed consistent performance under deliberate variations in chromatographic conditions. The specificity study verified the method’s ability to resolve the active ingredients from degradants and impurities, supporting its stability-indicating capability. The determined LOD and LOQ values, supported by both signal-to-noise ratios and statistical calculations based on standard deviation and calibration slope, highlight the method’s sensitivity and suitability for detecting and quantifying low levels of Brimonidine Tartrate and Timolol Maleate. The three complementary green analytical chemistry (GAC) metrics were applied and confirmed that the proposed RP-HPLC method possesses several environmentally favorable features such as simplicity, short analysis time, and low energy consumption. Overall, this validated method meets ICH and USP guidelines and offers a comprehensive analytical tool for reliable quality control, routine analysis, and stability testing of Combigan® and similar ophthalmic formulations. It thus represents a significant advancement toward ensuring the safety, efficacy, and quality of combination therapies used in glaucoma management.

## Data Availability

The original contributions presented in the study are included in the article/supplementary material, further inquiries can be directed to the corresponding author.

## Abbreviations

ACN, acetonitrile; Avg, average; FDA, Food and Drug Administration; HCl, hydrochloric acid; ICH, International Conference on Harmonisation; pKa, ionization constant; LC, liquid chromatography; LOD, limit of detection; LOQ, limit of quantitation; μL, microliter; μg/mL, microgram per milliliter; min, minutes; N, normality; Mg, milligram; ppm, parts per million; C18, column having octadecyl chain of carbon atom; pH, negative logarithm of H+ concentration; RP-HPLC, reverse-phase high-performance liquid chromatography; RSD, relative standard deviation; RT, retention time; R2, correlation of coefficient; UV, ultraviolet; GAC, green analytical chemistry; GAPI, Green Analytical Procedure Index.
